# Cofactor engineering improved CALB production in *Pichia pastoris* through heterologous expression of NADH oxidase and adenylate kinase

**DOI:** 10.1371/journal.pone.0181370

**Published:** 2017-07-17

**Authors:** Charumathi Jayachandran, Balakumaran Palanisamy Athiyaman, Meenakshisundaram Sankaranarayanan

**Affiliations:** Centre for Biotechnology, Anna University, Chennai, India; Universite Paris-Sud, FRANCE

## Abstract

The cofactor engineering strategy can relieve the metabolic stress induced by expression of recombinant protein in cellular metabolism related to cofactor and energy reactions. To study the effect of cofactor regeneration on recombinant protein expression, NADH oxidase (noxE) was engineered in *P*. *pastoris* expressing lipase B (GSCALB). Expression of noxE in *P*. *pastoris* (GSCALBNOX) increased NAD^+^ levels by 85% with a concomitant reduction in NADH/NAD^+^ ratio of 67% compared to GSCALB. The change in the redox level positively influenced the methanol uptake rate and made 34% augment in CALB activity. The decline in NADH level (44%) by noxE expression had lowered the adenylate energy charge (AEC) and ATP level in GSCALBNOX. In order to regenerate ATP in GSCALBNOX, adenylate kinase (ADK1) gene from *S*. *cerevisiae* S288c was co—expressed. Expression of ADK1 showed a remarkable increase in AEC and co—expression of both the genes synergistically improved CALB activity. This study shows the importance of maintenance of cellular redox homeostasis and adenylate energy charge during recombinant CALB expression in *P*. *pastoris*.

## Introduction

*Pichia pastoris* is a versatile host for expression of recombinant proteins that has been widely used for industrial applications. It holds an advantage over other expression systems due to the ease of well—established techniques for genetic engineering, high cell density cultivation and superior posttranslational modifications [1, 2]. Being methylotrophic yeast, *P*. *pastoris* provides an excellent pathway to metabolise methanol in a specialised compartment called peroxisome. Several studies about metabolic engineering in *P*. *pastoris* portrayed the regulatory aspects of intracellular metabolic reactions and development in recombinant protein production [3, 4]. In these, it was reported that the metabolic stress response against overexpression of recombinant protein redirects the intracellular carbon metabolism towards precursor formation to satisfy the increased energy demand. In addition to this, methanol oxidation stress results in accumulation of formaldehyde and hydrogen peroxide. Accumulation of these toxic compounds inhibits cell growth, protein synthesis and methanol consumption [5]. Furthermore, high gene dosages can induce unfolded protein responses that affect recombinant protein production. To overcome these limitations, overexpression of formaldehyde detoxifying enzymes like formaldehyde dehydrogenase (FLD), dihydroxyacetone synthase (DAS1) and folding chaperones [6, 7], expression at the lower (20°C) temperature [8] and optimisation of methanol feeding rate [9] were employed to minimise the toxic compounds accumulation, improve methanol metabolic reactions and recombinant protein production. Besides these efforts, the limitation in cofactor (NAD^+^, NADH, NADPH and ATP) availability is also one of the critical factors which interrupt the metabolic reactions and negatively influence recombinant protein synthesis [10].

The reducing cofactor NADH formed in cytosol was oxidised to NAD^+^ in mitochondria via electron transport chain (ETC), and the malate shuttle system helps in transportation. The maximum energy was generated from NADH oxidation in the respiratory chain and the ATP generated from this was consumed for cellular maintenance energy, heterologous protein synthesis and biomass build—up [11, 12]. Nie *et al*. reported that high expression of β- galactosidase (glucose medium) affected the NADH metabolism in *P*. *pastoris* [12]. This led to increasing NADH—dependent glycerol formation to maintain the cytoplasmic redox balance and restore the NAD^+^ level. Similarly, Jones *et al*. reported that metabolic response against the diauxic shift from glycerol to methanol and high gene dosage expression could affect the cytoplasmic—mitochondrial redox imbalance (recycle of NAD^+^) that results in the translational arrest [13]. The increase in NADH/NAD^+^ ratio by limitation in NADH recycling hinders the glycolysis and TCA cycle reaction [14]. The metabolic stress responses against the redox imbalance are overcome by the production of fermentative products like glycerol/ethanol in crabtree—positive yeast [15]. In crabtree—negative yeast like *P*. *pastoris*, secreted arabitol (NADPH—dependent YDL124WP) in response to oxidative stress and was predicted to be involved in redox balancing [16]. In—depth knowledge about engineering cofactor regeneration reaction for maintenance of the intracellular redox could help to understand the relationship between cofactor availability and recombinant protein expression.

**Fig 1:**
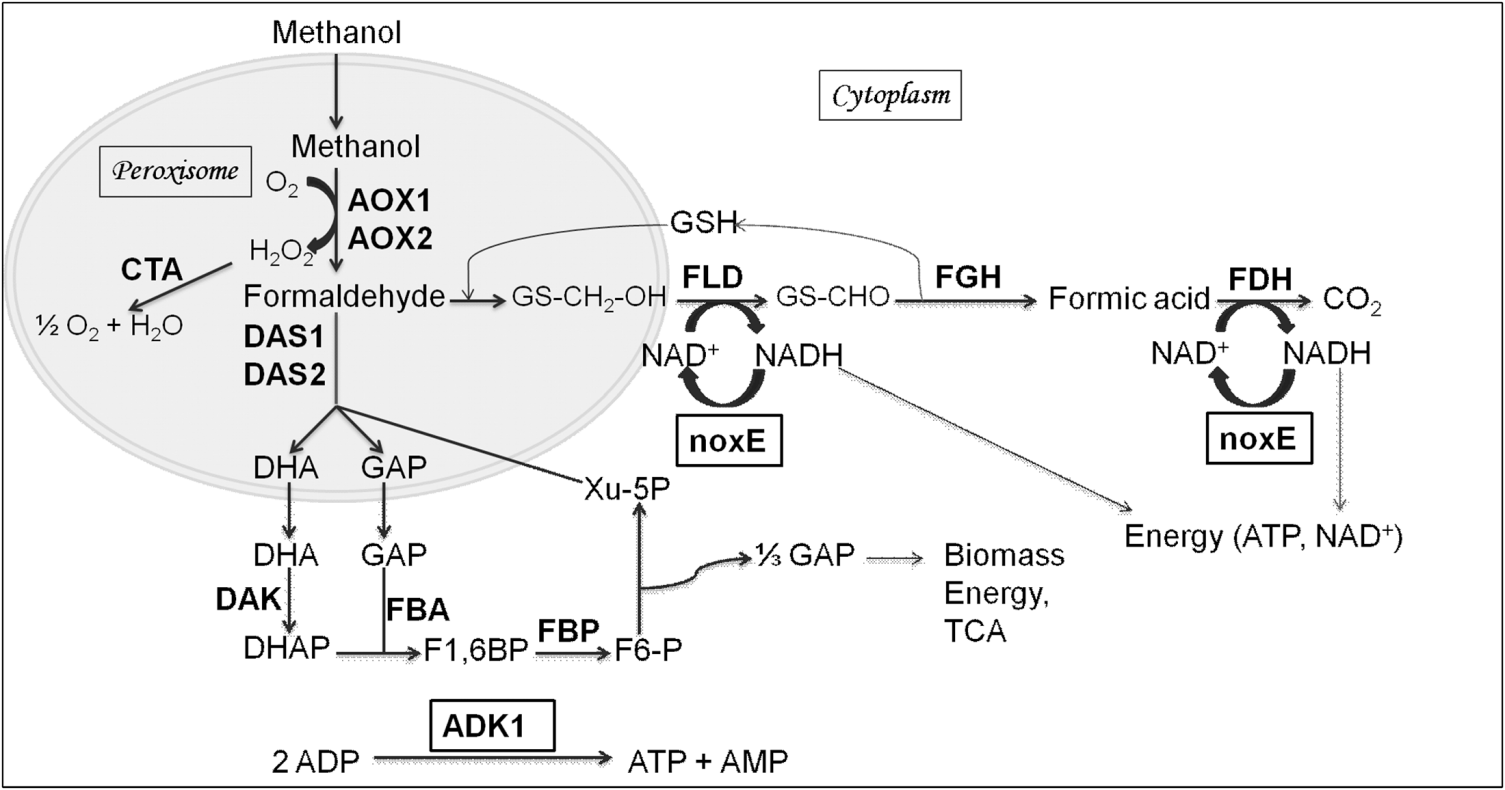
Methanol utilisation pathway of *P*. *pastoris* highlighting the possible modes of regeneration of cofactors. AOX1/2 —Alcohol oxidase, FLD—formaldehyde dehydrogenase, CTA—catalase, FDH—formate dehydrogenase, DAS1/2 —dihydroxyacetone synthase, DAK—dihydroxyacetone kinase, FGH—s-formylglutathione hydrolase, FBA— 1,6 —bisphosphate aldolase, FBP—Fructose 1,6 bisphosphatase, noxE—NADH oxidase, ADK1 —adenylate kinase, DHA—dihydroxyacetone, GAP—glyceraldehyde 3 —phosphate, DHAP—dihydroxyacetone phosphate, GSH—glutathione, Xu— 5 P—xylulose 5—phosphate, F1,6BP—fructose 1,6, bisphosphate, F6-P—Fructose 6 –phosphate.

To investigate the effect of cofactor regeneration in recombinant CALB expression and methanol metabolism, NADH oxidase from *L*. *lactis* was engineered in *P*. *pastoris* expressing CALB (GSCALB). noxE is a water forming enzyme which catalyses the oxidation of NADH to NAD^+^ and water. Several groups have reported in other microorganisms that engineering noxE efficiently regenerated NAD^+^, reduced the redox imbalance caused by NADH accumulation and improved production of industrial products [17–20]. Interestingly, engineering noxE also enhanced recombinant β—galactosidase expression in *E*. *coli* with a simultaneous reduction in redox ratio and acetate accumulation [21]. Our approach is first of its kind to report the progress in cellular metabolism of *P*. *pastoris* through cofactor regeneration enable an increase in expression of CALB. In addition, to compensate the decrease in adenylate energy charge due to the change in redox metabolism, adenylate kinase gene (ADK1) was co—expressed with noxE to enhance ATP regenerating efficiency for cellular energy maintenance. Heterologous expression of ADK1 gene from *S*. *cerevisiae* in *C*. *boidinii* improved ATP productivity when cultivated in medium supplemented with adenosine [22]. Increased ADK1 gene dosage in the same strain enhanced the adenylate kinase activity and ATP production [23]. The schematic representation of engineered *P*. *pastoris* used in this study was displayed in Fig 1. In the present research, the ADK1 gene from *S*. *cerevisiae* S288c was co—expressed with noxE in GSCALB to allow a possible regeneration of both NAD^+^ and ATP to enhance recombinant CALB activity.

## Materials and method

### Strains, vectors and media

*P*. *pastoris* GS115 (Invitrogen, USA) was used as the yeast expression host, and *E*. *coli* DH5α was used as the host for vector manipulations. Recombinant pPICZαB vector containing *Candida antarctica* lipase B (CALB) gene [24] expressed in *P*. *pastoris* GS115 with high copy CALB (GSCALB) was used in this study for protein expression analysis. *Lactococcus lactis* (subspecies: *Cremoris* MG1363) and *S*. *cerevisiae* S288c was used for amplification of noxE and ADK1 gene, pPIC6A vector and pPIC3.5K vector (Invitrogen, USA) were used as the cloning vector. Recombinant *E*. *coli* strains were grown in low—salt LB medium (1% tryptone, 0.5% peptone, 0.5% Sodium chloride) at 37°C at 200 rpm. *P*. *pastoris* was cultivated in YPD/YPG medium (1% yeast extract, 2% peptone, 2% dextrose/1% Glycerol) at 28°C at 200 rpm. BMMY (1% yeast extract, 2% peptone, 100 mM potassium phosphate pH 6.0, 1.34% Yeast Nitrogen Base, 4*10^−5^% biotin, 0.5–1% methanol) was used as the expression medium.

### Construction of *P*. *pastoris* GSCALB expressing noxE and ADK1

Primers used for amplification were listed in Table 1. The noxE gene was amplified from *L*. *lactis* MG1363 and inserted in pPIC6A vector under the control of the *AOX1* promoter. Similarly, ADK1 gene was amplified from *S*. *cerevisiae* S288c and inserted under the *AOX1* promoter in a pPIC3.5K vector. The recombinant vectors were linearised with SacI enzyme to enable integration in the genome at the *AOX1* site. GSCALB was transformed with 5–10 μg of linearised vector by electroporation at 1500 Volts, 250 Ω resistance and 50 microfarad capacitance (BTX Electroporator, USA). The colonies appeared in petri plates were confirmed for gene integration by PCR using gene specific primers. Both the enzymes were expressed intracellularly in GSCALB. GSCALB expressing noxE referred as GSCALBNOX, ADK1 as GSCALBADK and GSCALBNOX expressing ADK1 referred as GSCALBNOXADK.

**Table 1:** List of primers used in the study.

Gene name	Primer sequence (5’…. 3’)
noxE	Forward: CCG**CTCGAG**ATGAAA ATCGTAGTTATCGG
	Reverse: CTG**GGGCCC**TTATTTGGCATTCAAAGCTGC
ADK1	Forward: CTG**GGGCCC**ATGTCTAGCTCAGAATCCATTAG
	Reverse: CCG**CTCGAG**TTAATCCTTACCTAGCTTG
	**Primers used for real—time PCR**
	**Housekeeping gene**
qACT1	Forward: AGTGTTCCCATCGGTCGTAG Reverse: GGTGTGGTGCCAGATCTTTT
	**Methanol metabolism genes**
qAOX1	Forward: GAAGCTGCCCTGTCTTAAACCTT Reverse: CAAAAGCTTGTCAATTGGAACCA
qFLD	Forward: TGGATTATCTGTCATCCAAGGTGCAGTTTC Reverse: GTCCGCCCATGCCTTCTTTGAATC
qFDH	Forward: GTATTAGACAATGGCTTGAGAA Reverse: GGATGGAATGGAGTGGAA
qDAS1	Forward: CTGAGAAACCAGCTAAAGGTGACGAGT Reverse: TCTTGTCCCTCACGAGGGTACTCT
qCTA	Forward: CCACCTTCGACAACGCTAA Reverse: GGCAGCACAAACGTCAGAT
	**Glycolysis genes**
qGAPDH	Forward: ATGACCGCCACTCAAAAGAC Reverse: GCACCAGTGGAAGATGGAAT
qPYK	Forward: CAAGTGCAATCTGGCAGGTA Reverse: GCATCATAGCAACAGCCTCA
	**TCA cycle genes**
qCIT	Forward: TTCAAAGACGGAAAGGTTGG Reverse: CAACCAAGTGAGTGGTGTGG
qαKGDH	Forward: GCACTCTTCTGCCCGTTTA Reverse: AATGGGTGACGCAACAACT
qMDH	Forward: TGTCGTTGAGCCATCTTTCGTC Reverse: TGGCAGTTGTGATCATCTCCTCTT
	**Glutamate metabolism genes**
qGDH	Forward: TTGCCAATGCTGAGAGTGAG Reverse: AGCGATGGAGAGATCGTTTG
qGS	Forward: TGCGTGCTTTCTATGAGTGG Reverse: CAGATTGGCCCCACAATAAC

### Shake flask experiment studies

Yeast pre-culture was grown in 3 ml YPG medium inoculated with 50 μl glycerol stock and incubated at 28°C for 48 h. From the pre-culture, 1 ml was inoculated into 50 ml YPG medium in a 500 ml conical flask. Mid-log phase cells were harvested, and biomass was transferred to 10 ml BMMY medium. Cells were induced with 0.5% from 100% methanol (v/v) on the first day (24 h) and later 1% (v/v) for every 12 h. The samples were analysed for 6 days after methanol induction for recombinant protein expression. All the expression studies and analyses were conducted in triplicates.

### Analysis

CALB activity was assayed by pH—stat method using tributyrin as a substrate. The assay was performed using Titration manager (Radiometry, France). The reaction mixture contained 5 ml of 10% tributyrin, 2% gum arabic (as an emulsifier) and 80 mM sodium hydroxide (NaOH) for maintaining pH at 7. Culture supernatant harvested from shake flask after methanol induction was centrifuged at 10,000 rpm for 10 min. The reaction was started by the addition of 20 to 50 μl of clear culture supernatant. The enzyme activity was calculated from the volume of NaOH added with respect to time. The enzyme activity was expressed in units/ml. One tributyrin unit is defined as the amount of enzyme that liberates 1 μmol fatty acid per minute.

For residual methanol analysis, the culture supernatant was centrifuged at high—speed to remove debris and filtered through 0.2—micron size membrane. The supernatant was analysed for residual methanol by high—performance liquid chromatography using Aminex 87XH column (Bio—Rad) with mobile phase 5 mM sulphuric at flow rate 0.6 ml/min.

For intracellular enzymes assay, the cells harvested from the shake flask experiment were diluted to 5 OD_600nm_ in 50 mM potassium phosphate buffer (pH 7.0). The cells were washed twice with potassium phosphate buffer and centrifuged at 10,000 rpm for 5 min. About 0.5 ml cells were lysed by sonication at 4°C and centrifuged at 10,000rpm at 10 min to remove the cell debris. The formaldehyde dehydrogenase assay was performed as previously described [25]. The total protein concentration was quantified by Bradford’s method with BSA as standard.

For all intracellular nucleotide analysis, cells harvested from shake flask after methanol induction were quickly quenched in extraction buffer and frozen in liquid nitrogen. The frozen samples (5 OD_600nm_) in 0.4N perchloric acid were used for extraction of ATP, ADP and AMP. The perchloric acid extract was frozen and thawed three times and centrifuged at 14000 rpm for 5 min. The supernatant was neutralised to pH 7.2–7.5 with 4M potassium carbonate and incubated in ice for 10 min and centrifuged at 14000 rpm for 5 min. The supernatant was analysed by high—performance liquid chromatography using YMC—C18 Hydrosphere column with mobile phase 0.1 M potassium phosphate buffer (pH 5.5) at a flow rate 1 ml/min. The nucleotides were detected in UV detector (Agilent) at 260 nm wavelength. The concentration of each adenylate nucleotide was determined from the standard graph (50 to 500 μM). Adenylate energy charge was calculated from the formula (ATP + 0.5 ADP) / (ATP + ADP + AMP) [26]. The NAD^+^ and NADH nucleotide levels were determined by enzyme cycling method. The NAD^+^ and NADH were extracted separately in acidic solution (0.2M HCL) and alkaline solution (0.2M NaOH). The frozen samples (0.1 OD_600nm_) were incubated in water bath at 60°C for 10 min. The cells were immediately cooled to 0°C and neutralised with 0.1M NaOH for NAD^+^ and 0.1M HCL for NADH. The samples were centrifuged at 10,000rpm for 1 min. The supernatant was assayed for NAD^+^ and NADH nucleotide levels using enzyme cycling method [27].

### Real-time PCR analysis

The primers used for real—time PCR are listed in Table 1. Cells harvested from shake flask after methanol induction were diluted to 5 OD_600nm_ and processed for RNA extraction by homogenisation using liquid nitrogen. The total RNA was extracted from the cell lysate using the RNeasy mini kit (Qiagen). Residual genomic DNA was removed by DNase (Fermentas, USA) treatment. The DNase-treated RNA was purified and processed for cDNA synthesis by Superscript III Reverse Transcriptase kit (Invitrogen, USA). The concentration of nucleic acids was quantified in NanoDrop (Thermo Scientific, USA). Real—time PCR was analysed in StepOne real—time PCR system (Applied Biosystems) using Power SYBR green master mix (Invitrogen, USA). The C_t_ of the unknown was normalised with internal housekeeping gene beta—actin (ACT1). Gene expression was analysed by relative quantification method [28].

## Results

### Effect of NAD^+^ regeneration in intracellular redox ratio and CALB activity

**Fig 2:**
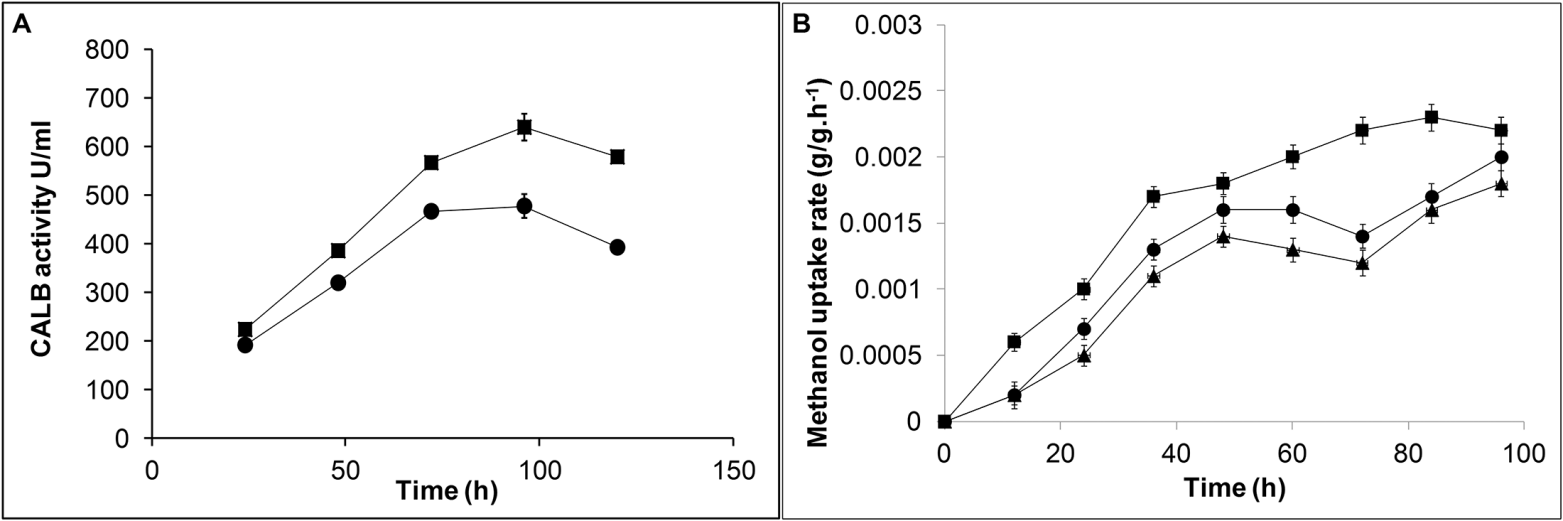
Effect of noxE expression on CALB activity and methanol uptake rate. A: The CALB activity was analysed after methanol induction. B: Methanol uptake rate determined at every 12 h time intervals. GS115 host (▲), GSCALB (●) and GSCALBNOX (■). The error bars represents the deviation from the mean.

In an attempt to investigate the effect of redox perturbation in intracellular metabolic reactions and recombinant CALB expression, noxE from *L*. *lactis* was expressed in *P*. *pastoris* GSCALB. The integration and expression of noxE in GSCALBNOX were confirmed by PCR amplification of the gene from genomic DNA and enzyme activity. All GSCALBNOX strains confirmed by PCR experiment showed higher CALB activity compared to GSCALB. Fig 2A illustrates the CALB activity achieved in GSCALBNOX strain having the highest activity compared to GSCALB. The expression of noxE enhanced the CALB activity from 478U/ml (in GSCALB) to 640U/ml in GSCALBNOX. This clone was further used in this study for comparative analysis with GSCALB.

**Fig 3:**
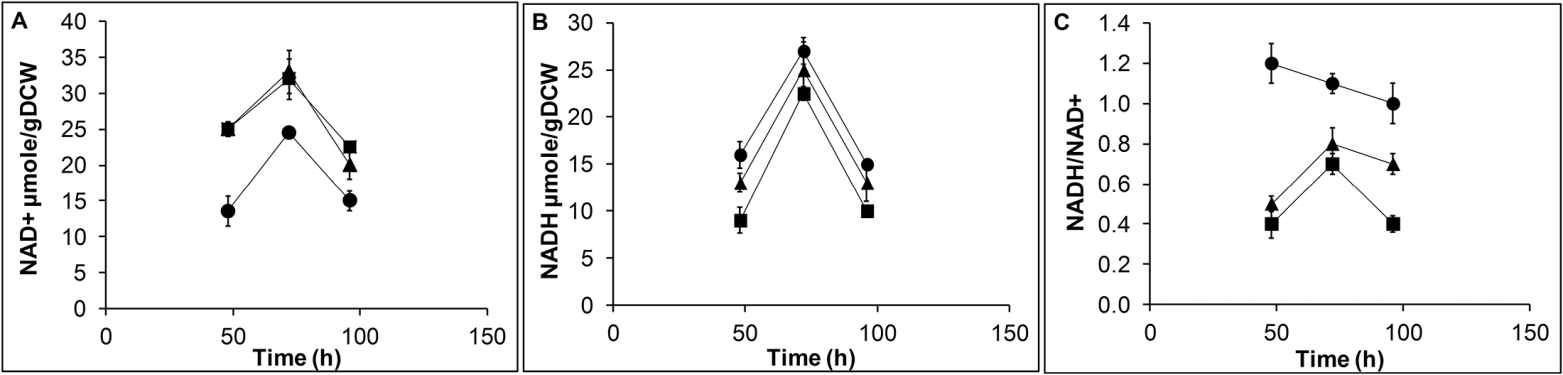
Effect of noxE expression on intracellular nucleotide levels. The intracellular nucleotide levels were determined in GS115 host (▲), GSCALB (●), GSCALBNOX (■) at three—time points 48, 72 and 96 h. The level of NAD^+^ (A), NADH (B) and NADH/NAD^+^ (C) was determined by enzyme assay. The nucleotide analysis was carried out in triplicates and the error bar represents the deviation from the mean.

The influence of methanol oxidation and CALB overexpression in cellular redox state was evaluated from intracellular pyridine nucleotide levels. The results were compared with GS115 host (referred as host strain) induced with methanol. Compared to host strain, both the strains GSCALB and GSCALBNOX had a similar growth rate in methanol medium. However, the difference in methanol uptake rate and intracellular redox ratio NADH/NAD^+^ of GSCALB compared to host strain was significant. The methanol uptake rate in GSCALB was higher than the host strain, but after 60 h the methanol consumption was lowered (Fig 2B). The NADH level and the redox ratio NADH/NAD^+^ was also higher in recombinant GSCALB strain compared to host strain (Fig 3). The maximum CALB activity obtained from GSCALB strain was 478U/ml at 96 h. Engineering noxE in GSCALBNOX produced a notable change in redox ratio, CALB activity and methanol uptake rate compare to GSCALB. Expression of noxE in GSCALBNOX resulted in 85% increase in NAD^+^ level and 44% decrease in NADH level compared to GSCALB (Fig 3A and 3B). The significant changes in the pyridine nucleotide concentration caused 67% reduction in NADH/NAD^+^ ratio which was lesser than both GSCALB and host strain (Fig 3C). The increase in NAD^+^ availability allowed GSCALBNOX to consume more methanol, which in turn influenced the CALB expression (Fig 2). The CALB activity was enhanced to 640U/ml at 96 h of post methanol induction, which corresponds to 34% augment in CALB activity in GSCALBNOX.

### Relative expression analysis to understand the influence of NAD^+^ regeneration in methanol pathway genes

**Fig 4:**
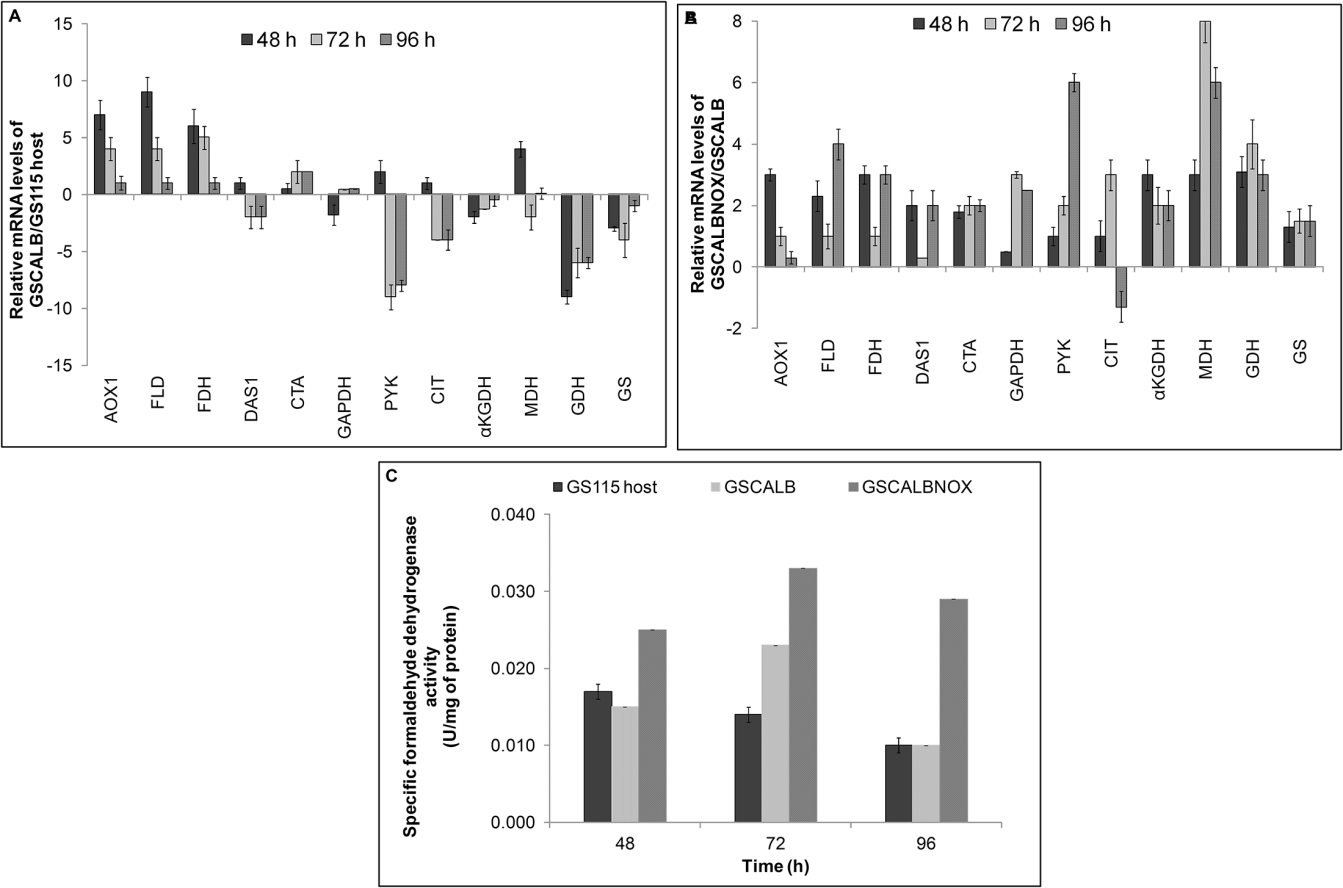
Comparison of relative gene expression levels of key metabolic genes and specific formaldehyde dehydrogenase enzyme activity in GSCALB and GSCALBNOX. Transcription of genes involved in methanol metabolism, glycolysis, TCA and glutamate catabolism were analysed by real- time PCR. The analysis was performed at 48, 72 and 96 h time intervals and the value represents the log_2_ fold change calculated from three biological experiments. A: Relative expression of GSCALB compared to GS115 host, B: Relative expression of GSCALBNOX compared to GSCALB, C: Intracellular specific formaldehyde dehydrogenase enzyme activity.

The increase in NAD^+^ regeneration and the methanol uptake rate may influence the transcription of methanol pathway genes. Initially, the impact of CALB expression in methanol pathway genes was observed in GSCALB compared to host strain. The transcription of alcohol oxidase (AOX1), formaldehyde dehydrogenase (FLD), formate dehydrogenase (FDH) and catalase (CTA) showed a significant increase in GSCALB. Glycolytic genes such as glyceraldehyde 3 –phosphate dehydrogenase (GAPDH) and pyruvate kinase (PYK) and TCA cycle genes like citrate synthase (CIT), alpha—ketoglutarate dehydrogenase (αKGDH) and malate dehydrogenase (MDH) were downregulated in GSCALB (Fig 4A). Expression of noxE showed a further increase in the transcription of methanol pathway genes compared to GSCALB (Fig 4B). The relative expression of AOX1 was 3 fold maximum at 48 h and later decreased to 0.3 fold at 96 h after methanol induction. Transcription of FLD and DAS1 genes responsible for detoxification of formaldehyde was upregulated. The increase in the CTA transcript level could be the effect of enhanced hydrogen peroxide produced from methanol oxidation. All the key enzymes of methanol pathway maintained their mRNA level above the threshold. Interestingly, genes related to glycolysis and TCA cycle were upregulated in GSCALBNOX. The mRNA levels of GAPDH increased to maximum 3 fold and PYK (generates ATP) increased from 1 fold to 6 fold. The relative expression of CIT, αKGDH and MDH (mitochondrial) were also upregulated. Glutamate catabolic genes (NAD^+^ dependent—glutamate dehydrogenase and glutamate synthase) which are assumed to produce 2—oxoglutarate to enter TCA cycle for energy synthesis, were also upregulated.

### Intracellular formaldehyde dehydrogenase enzyme activity

The enzyme assay was performed to validate the effect of increased intracellular NAD^+^ availability and gene expression profile of GSCALBNOX. Since formaldehyde dehydrogenase activity is the limiting factor in methanol metabolism, determination of its enzyme activity would indicate the key role of regenerating NAD^+^ in the biochemical reactions. Accordingly, the formaldehyde dehydrogenase enzyme activity was higher in GSCALBNOX than GSCALB (Fig 4C).

### Cloning and expression of ADK1 in GSCALBNOX

NADH generated from methanol dissimilatory pathway is oxidized through the respiratory chain for energy synthesis. The electrons generated from the NADH oxidation create a proton-motive force that enhances ATP synthesis by F_0_F_1_-ATP synthase. The improved NADH oxidation in the cytoplasm by noxE expression limited the transfer of electrons from NADH to ETC for ATP synthesis. This was evident from the adenylate energy charge (AEC) value of GSCALBNOX (Table 2). The AEC of GSCALB was comparable to that of host strain. The AEC indicates the metabolically available energy stored in the adenylate pool [26]. The AEC in GSCALBNOX decreased by 16% and 22% compared to host strain and GSCALB. Genetic manipulation of ATP regeneration can fulfil the energetic demand and increase the cellular biological reactions. For this purpose, ADK1 from *S*. *cerevisiae* S288c was co—expressed in GSCALBNOX. GSCALB expressing ADK1 (GSCALBADK) was constructed to evaluate the individual effect of ADK1 expression in AEC and CALB activity. Integration of the ADK1 gene was confirmed by PCR with vector forward and gene—specific reverse primers. In ADK strains, the AEC increased to 0.6 ± 0.02 throughout the cultivation compared to the original strains which are having AEC less than 0.5 ± 0.03 (Table 3). Although CALB activity increased for both the strains, it reached a maximum of 722U/ml for GSCALBNOXADK. Thus, to summarise the combined effect of the lower redox ratio and improved ATP regeneration in GSCALBNOXADK further improved recombinant CALB production in *P*. *pastoris*.

**Table 2:** Adenylate energy charge of GS115 host, GSCALB and GSCALBNOX.

Time (h)	GS115 host	GSCALB	GSCALBNOX
**48**	0.48 ± 0.012	0.45 ± 0.01	0.38 ± 0.02
**72**	0.49 ± 0.02	0.47 ± 0.02	0.41 ± 0.01
**96**	0.51 ± 0.03	0.55 ± 0.00	0.43 ± 0.01

Comparison of adenylate energy charge determined in GS115 host, GSCALB and GSCALBNOX at time interval 48, 72 and 96h. The mean ± SD value calculated from three experimental values.

**Table 3:** Effect of ADK1 gene expression on adenylate energy charge and CALB activity.

	GSCALBADK		GSCALBNOXADK	
Time (h)	CALB activity U/ml	Adenylate energy charge (AEC)	CALB activity U/ml	Adenylate energy charge (AEC)
48	378 ± 11	0.64 ± 0.03	454 ± 6	0.60 ± 0.00
72	505 ± 5	0.66 ± 0.02	654 ± 8	0.61 ± 0.03
96	540 ± 10	0.64 ± 0.02	722 ±11	0.61 ± 0.01

The values are the arithmetic mean ± SD calculated from three biological experiments.

## Discussion

Engineering cofactor regeneration in *P*. *pastoris* GSCALB improved recombinant CALB activity. Most of the enzyme activities are regulated by the availability of cofactors such as NADH, NAD^+^, ATP to maintain the redox ratio and cellular energy. The metabolic stress related to methanol oxidation and overexpression of recombinant protein enhances the cellular ATP demand and affects the NADH metabolism in *P*. *pastoris* [12, 13]. This study demonstrates that expressing noxE and ADK1 in *P*. *pastoris* GSCALB enhanced CALB activity through regeneration of NAD^+^ and ATP in the cytoplasm.

From the experimental results, it was evident that CALB expression in GSCALB influenced NADH level and therefore increased the intracellular redox ratio NADH/NAD^+^ compared to host strain. The increase in redox ratio NADH/NAD^+^ was due to the effect of methanol oxidation and other metabolic activities [29]. The overexpression of CALB and increased redox ratio NADH/NAD^+^ did not influence any detrimental effect on cell growth rate. The gene expression of AOX1, FLD, DAS1 and CTA are regulated at transcriptional level by methanol [30] whose expressional increase in GSCALB correlates with the initial increase in methanol uptake rate compared to host strain. The decline in methanol utilisation in later stages of experiment influenced the relative gene expression reduction of AOX1, FLD, FDH, DAS1 and CTA. Similar to the earlier prediction about the repression of glycolysis genes and TCA cycle genes when shifted to methanol cultivation [31], the mRNA levels of GAPDH and PYK (belongs to the downstream part of glycolysis), CIT, αKGDH and MDH (belongs to TCA cycle) in GSCALB were downregulated in GSCALB. The discrepancy in formaldehyde dehydrogenase mRNA levels and enzyme activity suggest that there may be a cofactor imbalance or accumulation of formaldehyde. Despite the high level of NADH generated in GSCALB, the AEC was similar to host strain which was likely due to an imbalance in NADH and NAD^+^ concentration between cytoplasm and mitochondria.

Expressing noxE in GSCALBNOX induced a notable change in intracellular redox ratio and methanol utilisation rate. Interestingly, this effect caused by noxE expression resulted in augmentation of CALB activity in GSCALBNOX. Our results were analogous to previous reports stating expression of noxE in *S*. *cerevisiae* minimised the redox ratio and redirected the carbon flux to core metabolism instead of producing fermentative by- products [32, 33]. The increase in NAD^+^ availability in cytoplasm enhanced the methanol uptake rate that led to changes in the relative expression of methanol metabolic genes. Expression of genes at the transcriptional level gives the theoretical information about intracellular carbon metabolism. The metabolic changes induced by cofactor regeneration were interpreted from the expression of genes in GSCALBNOX compared to GSCALB. The methanol pathway genes which are upregulated in GSCALB were further increased in GSCALBNOX suggests an excess amount of methanol had entered the peroxisome and was oxidised efficiently with less accumulation of toxic compounds. Also, it was observed that the upregulation of methanol pathway genes in the GSCALBNOX sustained in the later stage of cultivation while these genes are suppressed in GSCALB.

In *P*. *pastoris* the maximum energy was generated from FLD and FDH activity while TCA cycle was used for amino acid synthesis. Thus, TCA cycle and glycolysis are repressed in methanol cultivation compared to glycerol as a carbon source [34]. In the present research, the increase in specific formaldehyde dehydrogenase enzyme activity indicated no limitation in NAD^+^ level and counter produced more NADH in GSCALBNOX. But, the decline in AEC compared to GSCALB suggests the amount of NADH entering the mitochondria for ATP synthesis was reduced due to NADH oxidase activity. The decrease in AEC indicated an elevation of catabolic reactions to synthesise ATP in order to maintain cellular energetic state [35]. As a result of this phenomenon, an upregulated TCA cycle genes compensated for the higher energy demand. Results supporting the claim were reported in *T*. *glabrata* expressing noxE in which the glycolysis and TCA cycle enzyme activity was enhanced to fulfil the energy demand [36]. Moreover, the CIT and GAPDH were tightly regulated by NADH, ATP and NAD^+^ cofactor levels [37, 38], a significant change in these cofactors level might induced the gene expression. Likewise, the upregulated expression of NAD^+^ dependent glutamate dehydrogenase and glutamate synthase allows glutamate to enter TCA cycle by converting to 2 –oxoglutarate and NADH.

Thus, the development in CALB production by noxE expression was explained from two reasons: the enhanced methanol uptake rate and the upregulation of TCA cycle in GSCALBNOX. The *AOX1* promoter activity is regulated by methanol consumption and influences the recombinant protein expression [9]. Enhanced methanol uptake rate could increase the CALB gene induction which was under the control of *AOX1* promoter. Krainer *et al*. stated that overexpression of FLD gene in recombinant *P*. *pastoris* MUT^S^ improved 2 –fold efficiency in conversion of methanol to horseradish peroxidase and *Candida antarctica* lipase B production [6]. Similarly, overexpression of DAS1 gene in the same strain resulted in 3 –fold increase in methanol to recombinant protein conversion efficiency but increased the process time [6]. So, the increased CALB activity could be the result of elevated transcription of methanol pathway genes in GSCALBNOX compared to GSCALB.

In *P*. *pastoris* during the methanol utilisation, repression of TCA cycle was observed. To enhance the TCA cycle metabolism a non—repressing carbon such as sorbitol was fed along with methanol during the induction phase to improve recombinant protein production [29]. Nocon *et al*. suggested that overexpression of MDH enhanced recombinant protein expression in *P*. *pastoris* [39]. From these studies, it was evident that enhancing the TCA cycle pathway improves recombinant protein production which was done by cofactor regeneration in our study.

However, NAD^+^ regeneration reduced the availability of NADH for ATP synthesis from respiratory chain. Plantz *et al*. reported earlier that genetic manipulation for intracellular energy synthesis could favour the cellular requirements and enhance the protein synthesis [40]. Heterologous expression of ADK1 in methylotrophic yeast enhanced ATP levels [41]. So we coupled an ATP regenerating reaction with NAD^+^ regeneration to enhance cellular energy and simultaneously CALB activity. In this study, we observed a similar change in AEC by expressing ADK1 in GSCALBADK and GSCALBNOXADK. Expression of ADK1 in both strains positively influenced CALB activity. Compared to GSCALBADK, the coupled expression noxE- ADK1 showed synergistic improvement in CALB expression. From this, we put forward that in a NAD^+^ regenerating system, ATP levels are reduced due to recycling of NADH which is required for ATP regeneration. Hence, to meet the energetic demand overexpression of ADK1 in this system resulted in enhanced protein production.

## Conclusion

The present work was intended to study the effect of cofactor regeneration in recombinant *P*. *pastoris*. Engineering NAD^+^ regeneration could effectively recycle cofactors that enhanced methanol uptake and CALB production. Further co—expressing the ADK gene synergistically improved cellular energy charge and CALB production. This work will also be useful in metabolic engineering studies in *P*. *pastoris*, where higher NAD^+^ cofactor regeneration is necessitated.
